# Phosphatidylethanolamine-binding protein 1 (PEBP1) mediates the regulatory role of microRNAs (miRNAs)-205-5p in degranulation and histamine release

**DOI:** 10.1080/21655979.2022.2080387

**Published:** 2022-05-29

**Authors:** Yuting Kuang, Binya Hu, Min Huang, Sijun Zhao, Xionghui Wu, Mengping Zhang, Zhong Xie

**Affiliations:** Department of Otorhinolaryngology Head and Neck Surgery, Hunan Children’s Hospital, Changsha, Hunan, China

**Keywords:** Allergic rhinitis, miR-205-5P, PEBP1, HMGB1, TLR4

## Abstract

miR-205-5p plays a vital role in the inflammation of allergic rhinitis (AR). The study is designed to investigate the effects and mechanism of miR-205-5p in AR in vivo and in vitro. An OVA-induced mice model and anti-DNP IgE-induced RBL-2H3 cell model were established. The pathological alterations in the nasal mucosa were evaluated by hematoxylin-eosin (HE) staining. IgE and histamine levels were detected by corresponding kits and the expressions of PEBP1, High mobility group box-1 (HMGB1) and Toll-like receptor 4 (TLR4) were detected by western blot. The association of miR-205-5p and PEBP1 was determined by dual-luciferase reported assay. β-hexosaminidase activity was to evaluate the degranulation of RBL-2H3 cell. The pathological injury of nasal mucosa was significantly improved by miR-205-5p inhibition compared to AR mice. Following the treatment of miR-205-5p inhibitor, the levels of helper T cell (Th1) cytokines, interleukin (IL)-2 and interferon-γ (IFN-γ) were increased, while the levels of Th2 cytokines, IL-4 and IL-13, as well as the levels of IgE and histamine were markedly decreased in AR mice. We further found that miR-205-5P inhibition induced increased expression of PEBP1 and decreased expressions of HMGB1and TLR4. In vitro, miR-205-5P was verified to bind to PEBP1. PEBP1 silencing led to the reverse of miR-205-5p effects on decreasing the levels of β-hexosaminidase activity and histamine, as well as the expressions of HMGB1 and TLR4 on anti-DNP IgE-induced RBL-2H3 cells. Our results indicate that miR-205-5P inhibition may ameliorate pathological injury via PEBP1. MiR-205-5P/ PEBP1 could be potential drug targets in AR

## Graphical abstract

**Figure:**
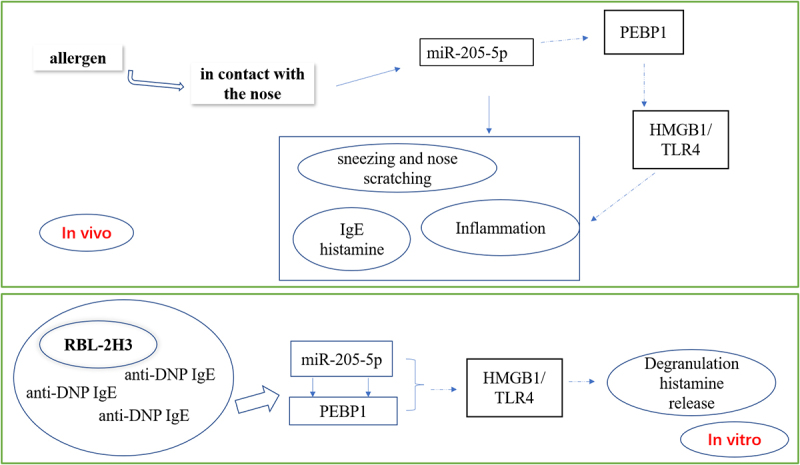

## Highlights

- The suppression of miR-205-5P possesses potential effects against allergic rhinitis.
- The regulatory role of miR-205-5P is mediated by targeting PEBP1 in allergic rhinitis.
- MiR-205-5P/PEBP1 modulates HMGB1/TLR4 pathway to involve in allergic rhinitis.

## Introduction

Allergic rhinitis (AR) is one of the most common allergic diseases in the world [1] Epidemiological study has shown that it affects 10% to 40% of the global population and the prevalence rate of AR in China is as high as 17.6% [2]. MicroRNAs (miRNAs) are endogenous single stranded non-coding RNAs with length of 18 to 22 nucleotides [3]. The treatment of allergic diseases is mostly aimed at alleviating symptoms. Despite more and more extensive research, there is still no established method to prevent and treat AR. At present, the main treatment for AR is glucocorticoid and other medications, and allergen-specific immunotherapy [4]. The former has a good effect in alleviating symptoms, but there are many adverse effects. The latter method is effective, but the disadvantage is that it takes a long time and is difficult to standardize due to the complex composition of the vaccine [5,6]. MiRNAs have opened up a new direction for the treatment of allergic diseases and are potential new biological target molecules with potential therapeutic effects in allergic diseases [7].

Current studies have noted that miRNA is associated with a variety of clinical diseases, including AR, chronic sinusitis, asthma, atopic dermatitis and other immune diseases [8–11]. A recent report demonstrated that miR‑205‑5p was involved in inflammatory response in AR with the help of B‑cell lymphoma 6 [12]. Previous research has found that PEBP1, also called RAF1 kinase inhibitory protein (RKIP1), can increase allergic responses in mast cells [13], indicating that PEBP1 was involved in AR. PEBP1, as a promiscuous small scaffolding protein, participates in AR by regulating ferroptosis via forming complexes with 15LO1 and 15LO2 to induce the generation of hydroperoxy-PE [14]. Additionally, a study found that PEBP1 caused the interaction between proferroptotic 15LO1 and the autophagic protein microtubule-associated light chain-3 to involve in asthma [15]. In addition to these regulatory roles of PEBP1, some studies suggest that PEBP1 can inhibit the expression of high-mobility group box 1 (HMGB1) signal, which is a ubiquitous nuclear protein to trigger inflammation in AR [16,17]. With this background, the study hypothesized that miR‑205‑5p and PEBP1 are engaged in the pathogenesis of AR and there could exist an association between miR‑205‑5p and PEBP1. The study focusing on the regulatory role of miR-205-5p not only shed light for further exploration of the pathomechanism of AR, but also provides a novel sight for the development and application of electrochemical biosensors, which has highly sensitive, facile, and low-cost as a method for the detection of miRNA markers [18,19]. With this background, this paper intends to explore whether the inhibition of miR-205-5p can target and regulate PEBP1 to inhibit the expression of HMGB1, and participate in the pathology of allergic rhinitis in vivo and in vitro, thereby elucidating the regulatory mechanism of miR-205-5p, which could provide novel sight for deeper research on the pathological mechanism of AR and recognize new targets for the treatment of AR.

## Method

Animal model

The BALB/c male mice (5 weeks, 20 g) were purchased from Charles River Labs (Beijing, China). The mice were fed for 7 days under standard laboratory conditions (23 ± 2°C, relative humidity of 55%±10%, light/dark cycles of 12 h) and feel free to get access to OVA-free food and water. On days 1, 8, and 15, mice were sensitized by intraperitoneal injection of 300 μL of mixture of OVA (50 μg) and 1 mg aluminum hydroxide dissolved in phosphate buffer (PBS). On day 22, mice received a daily OVA nasal challenge (10 mg/ mL, 20 μL/nostril) for 7 days. The control group was intragastrically given the same volume of normal saline. After the final OVA nasal challenge, the sneezing and nose scratching symptoms of each mouse within 30 minutes were recorded to investigate whether the modeling was successful. Symptoms were scored as described by previous study [20]. Blood samples were collected from mouse orbits. Serum was obtained by centrifugation at 4°C at 2000 × g for 10 min, and then stored at −80°C until use. The mice were sacrificed by Isoflurane anesthesia. All experiments were conducted according to the guidelines approved by the Animal Care and Use Committee of Hunan STA laboratory animal Co.,LTD (Hunan, China, Number: IACUC-SJA2022036).

RT-qPCR assay

Total RNA was extracted from Nasal mucosa tissue and RBL-2H3 cells using Trizol reagent (Invitrogen). For the analysis of the mRNA levels of PEBP1, HMGB1 and TLR4, total RNA was reverse transcribed into cDNA using RT Master Mix for qPCR strictly following manufacturer’s protocols (MedChemExpress LLC). Next, cDNA was amplified using TaqMan Fast Advanced Master Mix according to manufacturer’s guidance (ThermoFisher). GAPDH was used to be as an internal reference. For the detection of miR-205-5P, microRNA was subjected to reverse transcription using TaqMan MicroRNA Reverse Transcription Kit in accordance with manufacturer’s instructions. TaqMan MicroRNA Assay Kit was used to perform qRT-PCR reaction on Applied Biosystems 7500 Real-Time PCR System (ABI, USA). U6 was chosen as an endogenous control.

Hematoxylin-eosin (HE) staining and toluidine blue

The mice noses were fixed with 4% paraformaldehyde for 48 h. Then, the noses were decalcified in 20% EDTA Na decalcified solution for 2 weeks. After the decalcification was completed, the nose specimens were embedded with paraffin and cut into 5 μm thick sections. The sections were stained with HE and toluidine blue (for mast cells) solutions in line with the manufacturer’s instructions.

ELISA assay

Th1-related cytokines IL-2, IFN-γ and Th2-related cytokines IL-4 and IL-13 in the serum were detected using corresponding kits (mlbio, Shanghai, China) according to manufacturer’s guidance. In addition, the levels of IgE in mouse serum were detected by ELISA kit and histamine levels were also detected using HIS ELISA kit according to manufacturer’s procedures.

Western blot

The nasal mucosal tissues of mice were cut into pieces, ground, and lysed in RIPA lysis buffer containing protease inhibitors to obtain total proteins. Then, these proteins were subjected to centrifugation at 12,000 g for 10 min at 4°C. The protein concentration was determined by BCA kit (Beijing Solaibao Biological Technology Co., Ltd, Beijing, China). The equivalent amount of protein (20 μg) was separated by 10% sodium dodecyl sulfate-polyacrylamide gel electrophoresis, and then transferred to polyvinylidene fluoride membranes, which were blocked with TBST containing 5% skimmed milk for 2 h. The membranes were then incubated overnight with the primary antibodies at 4°C, followed by incubation with horseradish peroxidase-labeled goat anti-rabbit IgG secondary antibody at room temperature for 1 h. Finally, ECL chemiluminescence kit was used for color development of protein blots.

Cell culture

RBL-2H3 cells were purchased from Procell (Wuhan, China) and kept in culture in MEM medium containing 15% FBS at 37°C with 5% CO_2_. Anti-DNP IgE (1 μg/mL) was used to stimulate cells for 2 h and then cells were challenged with DNP-conjugated HSA (25 ng/mL) for 30 min.

Plasmid transfection

miR-205-5P mimic or its mimic NC were transfected into RBL-2H3 cells using Lipofectamine 2000 (ThermoFisher) in keeping with manufacturer’s guidance. After 24 h, the level of miR-205-5P was detected by quantitative real-time PCR (RT-qPCR). Before DNP IgE stimulation, cells were transfected with miR-205-5P inhibitor or its inhibitor NC, or in combination with small interference RNA (siRNA) targeting PEBP1 or its control group (siRNA-NC). These mimic, inhibitor and siRNA were purchased from RIBOBIO (Guangzhou, China).

Luciferase report assay

miR-205-5P mimic or its control mimic-NC, were transfected into RBL-2H3 cells together with reporter plasmids containing PEBP1 3’-UTR WT or 3’-UTR mutant sequences using Lipofectamine 2000 (Thermo Fisher). After transfection of 48 h, the luciferase activities were detected using Dual-luciferase Reporter Assay System (Promega, USA).

Degranulation and histamine release detection

Degranulation levels in RBL-2H3 cells were evaluated by β-Hexosaminidase Activity Assay by referring to a previous study [21] and the levels of histamine were detected by enzyme linked immunosorbent assay (ELISA) kit (ab213975, Abcam, England) according to manufacturer’s protocols.

Statistical analysis

The experimental data were shown in the form of mean ± standard deviation (SD). One-way ANOVA analysis was performed to compare the difference among multiple groups, followed by Tukey’s hoc test. p < 0.05 was considered statistically significant.

## Results

MiR-205-5P inhibitor ameliorated nasal mucous pathology induced by OVA

**Figure 1.**
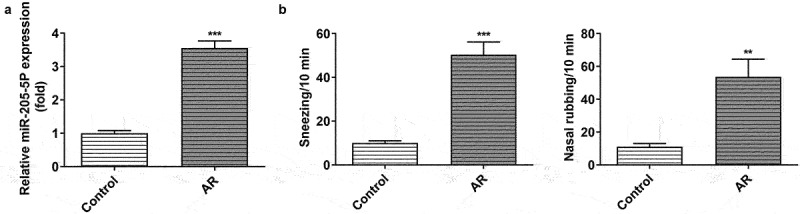
OVA-induction increased miR-205-5P level, sneezing and nose scratching. (a) The expression of miR-205-5P level. (b) The levels of sneezing and nose scratching. **P < 0.01, ***P < 0.001 versus control group.

**Figure 2.**
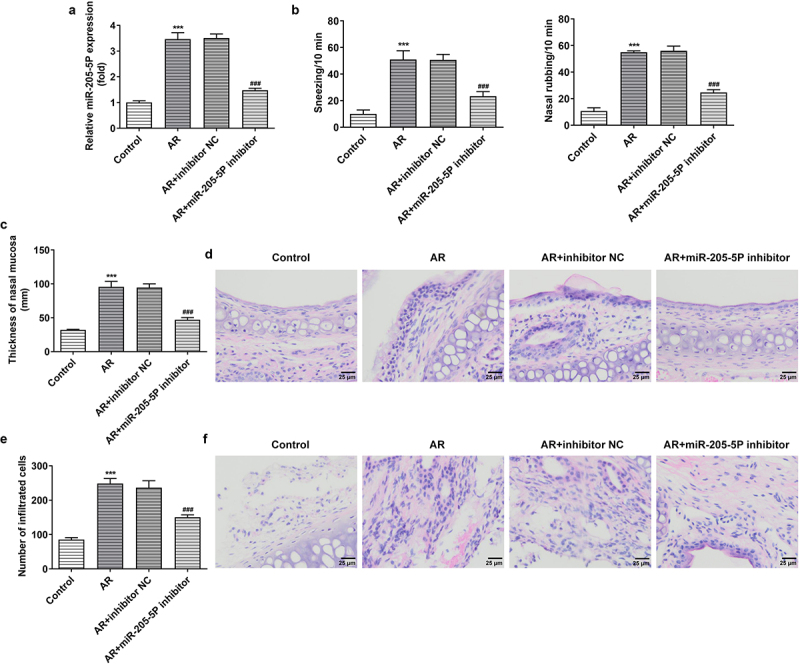
MiR-205-5P inhibitor improved allergic rhinitis. (a) The expression of miR-205-5P in nasal mucosa. (b) The levels of sneezing and nose scratching. (c) The thickness of nasal mucosa. (d) Histological analyses for AR nasals mucosa tissue. (e) Number of mast cell infiltration. (f) HE staining for analysis of mast cell infiltration. ***P < 0.001 versus control group, ^###^P < 0.001 versus AR + inhibitor NC.

**Figure 3.**
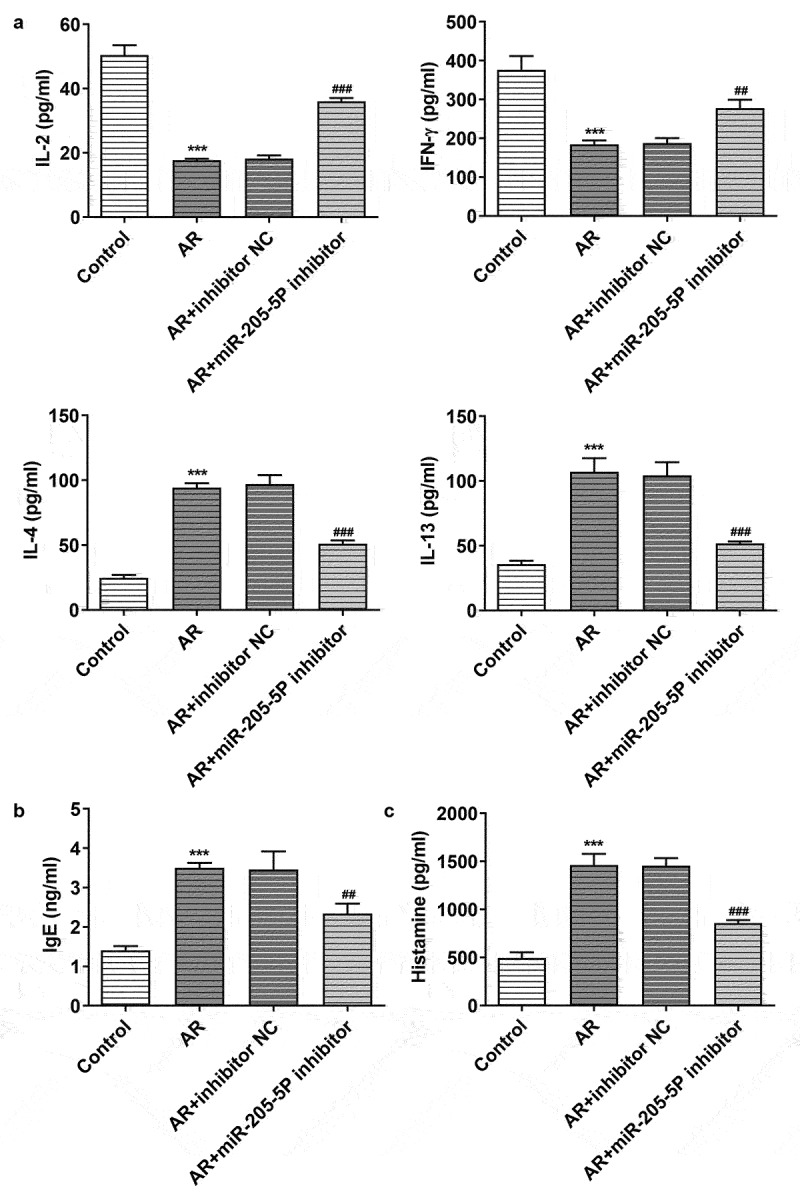
MiR-205-5P inhibitor modulated the levels of Th1/Th2 cytokines and decreased lgE and histamine levels in OVA-induced AR mice. (a) The levels of IL-2, IFN-γ, IL-4 and IL-13. (b) IgE level. (c) Histamine level. ***P < 0.001 versus control group, ^##^P < 0.01, ^###^P < 0.001 versus AR + inhibitor NC.

miR-205-5P has been reported to be involved in inflammatory response [22,23]. To analyze the association between miR-205-5P and AR, the role of miR-205-5P in OVA-induced mice with AR was assayed. OVA-treated mice showed increased expression of miR-205-5P in nasal mucous tissue (Figure 1(a)). The allergic nasal symptoms, sneezing and nose scratching, were markedly increased after OVA-administration in AR mice (Figure 1(b)). To further explore the role of miR-205-5P on AR, miR-205-5P inhibitor or NC (20 μL/mouse) were used at a rate of 1 μL/min before OVA challenge daily on days 22–28. It was apparently seen that miR-205-5P level was significantly reduced by miR-205-5P inhibitor (Figure 2(a)). The allergic nasal symptoms, sneezing and nose scratching in AR mice treated with miR-205-5P inhibitor were estimated. As can be seen from the data in Figure 2(b) that AR symptoms were reduced miR-205-5P inhibitor-treated AR mice compared to miR-205-5P-NC treated mice. Histological analyses for nasals mucosa tissue of AR mice showed that miR-205-5P inhibitor could reduce pathological changes and thickness of nasal mucosa in AR mice, as well as the number of mastocyte infiltration (Figure 2(c,f)). Treatment with miR-205-5P inhibitor contributed to increased IL-2 and IFN-γ levels in serum and decreased IL-4 and IL-13 levels in AR mice compared with that in AR mice with inhibitor NC treatment (Figure 3(a)). Besides, miR-205-5P inhibitor significantly decreased lgE and Histamine levels induced by OVA (Figure 3(b)).

miR-205-5P modulates the expression of PEBP1/HMGB1/TLR4

**Figure 4.**
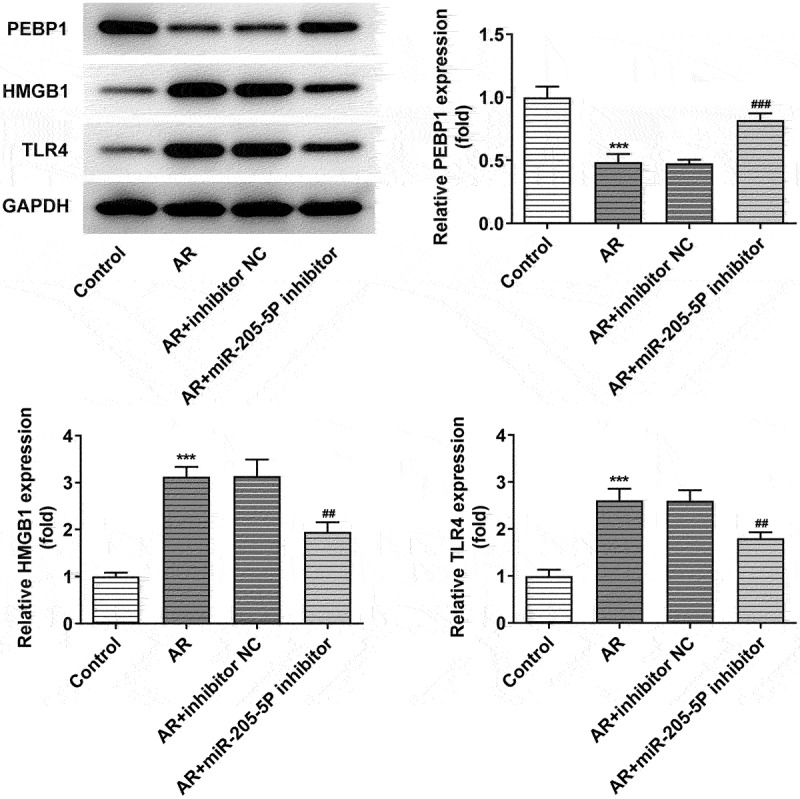
miR-205-5P inhibitor increased decreased PEBP1 expression and the expressions of HMGB1/TLR4. ***P < 0.001 versus control group, ^##^P < 0.01, ^###^P < 0.001 versus AR + inhibitor NC.

miR-205-5P was predicted to bind to PEBP1 through miRDB database (http://www.mirdb.org/). We attempted to investigate whether miR-205-5P could modulate the expressions of PEBP1/HMGB1/TLR4 in AR mice. PEBP1 expression was downregulated and the expressions of HMGB1 and TLR4 were upregulated in nasal mucosa of AR mice compared to control mice, while the treatment of miR-205-5P inhibitor significantly reversed this trend (Figure 4), suggesting that miR-205-5P could regulate PEBP1/HMGB1/TLR4.

MiR-205-5P binds to PEBP1

**Figure 5.**
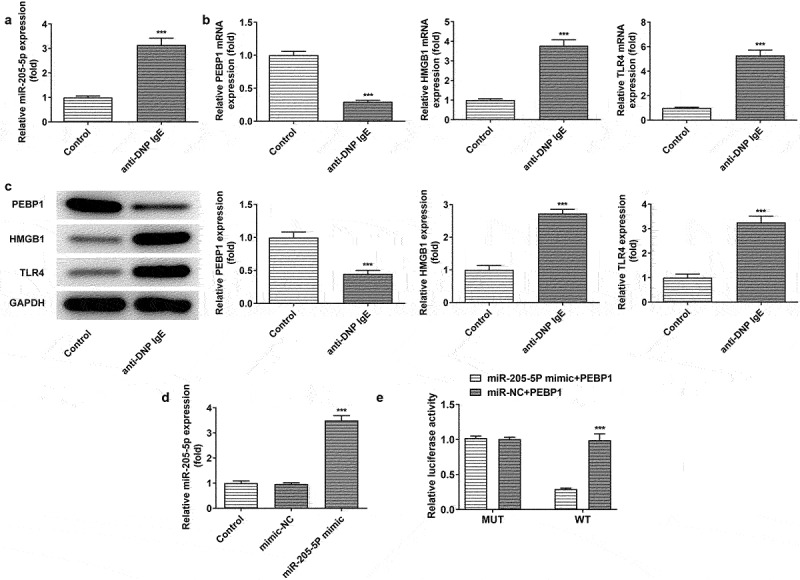
DNP IgE/HAS treatment induced the expression of miR-205-5P/PEBP1/HMGB1 in BL-2H3 cells. (a) The level of miR-205-5P. ***P < 0.001 versus control group. (b) The mRNA levels of PEBP1/ HMGB1/TLR4. ***P < 0.001 versus control group. (c) The protein levels of PEBP1/ HMGB1/TLR4. ***P < 0.001 versus control group. (d) The level of miR-205-5P. ***P < 0.001 versus mimic-NC. (e) The relationship of miR-205-5P and PEBP1 was detected by Luciferase report assay. ***P < 0.001 versus miR-205-5p mimic+PEBP1 + WT.

To further explore the mechanism of miR-205-5P in vitro model of AR, RBL-2H3 cells were stimulated with anti-DNP IgE for 24 h. The level of miR-205-5p was increased after anti-DNP IgE treatment while PEBP1 expression was decreased and the expressions of HMGB1 and TLR4 were increased (Figure 5(a,b)). After overexpressing miR-205-5p using miR-205-5p mimicmiR-205-5p expression was successfully elevated (Figure 5(d)). Dual luciferase reporting assay revealed that miR-205-5p could target PEBP1 (Figure 5(e)).

MiR-205-5P promotes degranulation and histamine release via PEBP1 in anti-DNP IgE-induced RBL-2H3 cells

**Figure 6.**
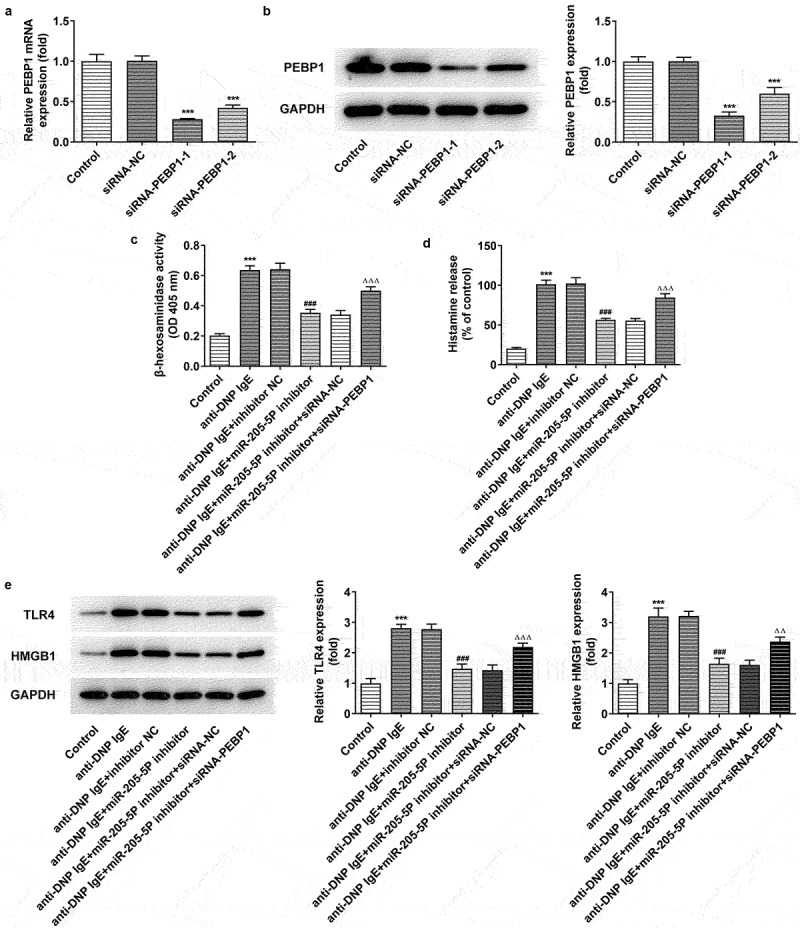
miR-205-5P/ PEBP1 regulated degranulation of BL-2H3 cells. (a) The mRNA level of PEBP1. (b) The protein level of PEBP1. ***P < 0.001 versus siRNA-NC. (c) The detection of β-hexosaminidase activity. (d) Histamine release level. (e) The expressions of TLR4 and HMGB1. ***P < 0.001 versus control group. ^###^P < 0.001 versus anti-DNP IgE + inhibitor NC. ^ΔΔ^P<0.01, ^ΔΔΔ^P<0.001.

To further explore the mechanism of miR-205-5P in DNP IgE-induced RBL-2H3 cells, we silenced the expression of PEBP1. It can be seen clearly PEBP1 expression was markedly declined following PEBP1 knockdown (Figure 6(a,b)). SiRNA- PEBP1-1 was chosen for subsequent experiments as siRNA-PEBP1-1 had better knockdown efficiency than siRNA-PEBP1-2. More importantly, anti-DNP IgE increased β-hexosaminidase activity and histamine release of BL-2H3 cells, but miR-205-5P inhibitor treatment could significantly reverse this effect, while PEBP1 silencing could markedly reverse the effects of miR-205-5P inhibitor (Figure 6(c,d)). We found that anti-DNP IgE treatment promoted the expressions of TLR4 and HMGB1 compared with the control group. miR-205-5P inhibitor resulted in the decrease of TLR4 and HMGB1 expressions, while PEBP1 silencing induced the increase of TLR4 and HMGB1 expressions again (Figure 6(e)).

## Discussion

In our work, we observed AR symptoms and pathological changes, such as sneezing, nasal scratching, elevated IL-4, IL-13 and IgE levels and declined IL-2 and IFN-γ levels in OVA-treated mice. Furthermore, there was a decrease in PEBP1 expression and an increase in HMGB1 and TLR4 expressions in OVA-induced AR. These impacts were significantly blunted by miR-205-5P inhibition. The results demonstrated that allergens can result in allergic diseases accompanied by alteration of PEBP1, HMGB1 and TLR4 levels via miR-205-5P. Additionally, in DNP IgE/HAS-induced RBL-2H3 cells, increased degranulation level and upregulation of HMGB1 and TLR4 expressions were observed in mast cells. This impact was significantly reversed by miR-205-5P inhibition, this influence of which was again restored by PEBP1 silencing, suggesting that miR-205-5P/PEBP1 regulated degranulation of mast cells in response to DNP IgE/HAS stimulation. It has been reported that the downregulation of HMGB 1/TLR4 pathway could attenuate AR via decreasing expression levels of inflammatory factors and the infiltration of lymphocyte and monocytes [24–26]. These findings illustrated that miR-205-5P/PEBP1 is involved in the pathogenesis of AR, this role of which could be related to HMGB 1/TLR4 pathway.

MiRNAs were discovered to be abnormally expressed in nasal mucus of AR patients compared with those healthy patients [27]. In our work, OVA-induced AR led to an upregulation of miR-205-5P in nasal mucus of mice. Moreover, miR-205-5P inhibition upregulated IL-2 and IFN-γ levels and downregulated IL-4 and IL-13 levels in AR mice, accompanied by a decrease in IgE and histamine levels. MiRNAs are reported to affect the development of allergic diseases by intervening Th1/Th2 polarization and facilitating chronic inflammation [28]. What’s more, Th2 cytokine, IL-4 could induce the subsets of Th2 cells and lead to IgE production and IL-13 is involved in maintaining IgE production and IgE-induced responses [29]. The results in our experiments suggested that increased miR-205-5P expression altered the levels of Th1/Th2 cytokines in OVA-induced AR, thereby affecting IgE production. Furthermore, it was reported that miR‑205‑5p knockdown improved AR by targeting BCL6 [12]. Based on the present study, miR‑205‑5p bound to 3UTR of PEBP1 and regulated its expression to involve in the pathogenesis of AR. Emerging evidences have noted that PEBP1 expression is regulated by various factors, such as specific proteins and some microRNAs [30–32]. We demonstrated that miR‑205‑5p modulated PEBP1 expression. OVA challenge or DNP IgE/HAS treatment contributed to a decrease in the expression of PEBP1 in our study. A large part of the literature has focused on the role of PEBP1 as a tumor suppressor [33–35], and indeed, PEBP1 also plays a vital role in the pathology of AR. A previous report found that PEBP1 expression was decreased in IgE−Fc receptor-Stimulated Mast Cells and in Peripheral Blood from Asthma Patients [13]. Therefore, PEBP1 could modulate allergic response and mast cell activation mediated by IgE−Fc receptor.

## Conclusion

Collectively, miR-205-5P inhibition significantly alleviated OVA-induced allergic nasal symptom in mice and decreased degranulation level of mast cells via PEBP1. Evidently, miR-205-5P/ PEBP1 plays a vital role in the pathogenesis of AR. The present study highlights the potential of targeting miR-205-5P/ PEBP1 as a therapeutic strategy for AR treatment.

## Data Availability

The datasets used and/or analyzed during the current study are available from the corresponding author on reasonable request.

## Limitations

The present study recognized the regulatory role of miR-205-5P/ PEBP1 involved in AR in vivo and vitro. However, the role of miR-205-5P/ PEBP1 in AR and whether targeting miR-205-5P/ PEBP1 is a potential therapy method against AR still needs to be further investigated in clinics.
